# Religious Wellbeing as a Predictor for Quality of Life in Iranian Hemodialysis Patients

**DOI:** 10.5539/gjhs.v6n4p261

**Published:** 2014-04-24

**Authors:** Zahra Taheri Kharame, Hadi Zamanian, Sahar Foroozanfar, Shirin Afsahi

**Affiliations:** 1 School of Paramedical Sciences, Qom University of Medical Sciences, Qom, Iran; 2 Health Policy Research Center, Qom University of Medical Sciences, Qom, Iran; 3 Department of Clinical Psychology, Science and Research Branch, Islamic Azad University, Karaj, Iran

**Keywords:** spiritual well-being, quality of life, hemodialysis patients

## Abstract

**Aim:**

Spiritual well-being is known as a main resource for adjustment and coping when confronted with stressful situations such as managing a chronic disease. The aim of this study is to determine the very relationship between spiritual well-being and quality of life in hemodialysis patients.

**Methods:**

A convenience sample of 95 patients with end-stage renal disease who were referred to main hemodialysis centers were included from December 2012 to June 2013. Data was collected by using a socio-demographic questionnaire, the SF-36 quality of life scale, and the spiritual wellbeing scale. Descriptive analysis, Pearson’s correlation and logistic regression analysis were performed for statistical assessment.

**Results:**

The mean age of the patients evaluated was 50.4 (SD=15.72) years of age, and 61.1% of the patients were male. Both religious and existential domains of spiritual wellbeing were associated with bodily pain, vitality, social functioning and mental health (P<0.05). The results of multiple logistic regression showed that religious well-being was associated with better quality of life in both domains of physical (OR=1.17; p=0.01) and mental (OR=1.14; p=0.02) components after controlling for socio-demographic and clinical variables.

**Conclusion:**

Religious well-being should be considered important predictive factors for the better quality of life in hemodialysis patients. This indicates the need for psychosocial and spiritual supports in the care of these patients.

## 1. Introduction

End-stage renal disease (ESRD) is an important health problem worldwide (Santos & Arcanjo, 2012; Bayoumi et al., 2013). In Iran, specifically the prevalence and incidence rates of ESRD have increased from 49.9 cases per million people in 2000 to 63.8 cases per million people in 2006, an almost 28% increase over a 6 year period (Pakpour et al., 2010). Patients with ESRD suffer from several physical and psychosocial complications and conventional treatment (hemodialysis) can impair quality of life (QoL) (Weisbord et al., 2005; Abdel-Kader et al., 2009). QoL is a multidimensional concept related to physical, mental and emotional, social functioning and spirituality (WHOQoL. 2006). Many people experience spirituality as an important support while trying to cope with a chronic illness or life-threatening disease (Vallurupalli et al., 2011). Spirituality is a quality that goes beyond religious affiliation and involves striving for inspiration, meaning, and purpose, irrespective of a belief in any God (Murray et al., 1985). A recent overview of measures of spirituality defined the term as ‘‘one’s striving for an experience of connection with oneself, connectedness with others and nature and connectedness with the transcendent’’ (de Jager Meezenbroek et al., 2012). One aspect of spirituality is spiritual wellbeing, the degree to which a person perceives or derives a sense of well-being from spiritual attitudes and strivings. Spiritual wellbeing is a barometer of how well a person copes with challenges and may play a role in health outcomes equal to or greater than spirituality it itself (Khanna et al., 2013).

Spirituality is experiencing transcendent meaning and purpose in life. Spiritual care could be effective in quality of life of patients suffering from cancer or other chronic diseases (Poder & Lemieux, 2013). Because of the nature of spiritual well-being which has two existential and religious dimensions, it could affect hopefulness and meaning of life/death in patients with chronic diseases such as cancer. As well, it could affect the coping styles which the patients use to encounter the stress of disease and by these psychological mediators changes a variety of indicators of mental health and quality of life (Jafari et al., 2014; Unterrainer et al., 2014; Dalmida et al., 2011; Rawdin et al., 2013).

In several studies spirituality has been consistently found to be an important factor affecting QOL in most chronic conditions. Following a literature review, Cotton et al. (1999) in their study among 142 women diagnosed with breast cancer reported spiritual wellbeing as correlated with both quality of life and psychological adjustment. In patients with chronic musculoskeletal pain, Rippentrop et al. (2005) found that spiritual experiences and religious support significantly predicted mental health status. In another study, Hammermeister et al. (2005) concluded that ‘‘spiritual well-being happens to have a positive influence on most aspects of health’’. Allahbakhshian et al. (2010) in their study among 236 patients, members of the Iran’s MS Society, reported a significant relationship between religious aspect of spiritual wellbeing and psychological aspect of quality of life. They also found a significant relationship between spiritual existential aspect of well-being and the both physical and moral aspects of quality of life. Also, a previous study conducted among Iranian elderly people residing in Kahrizak senior house showed that there was a positive correlation between spiritual wellbeing and quality of life (Jadidi et al., 2011). Davison et al. (2010) evaluated 253 hemodialysis patients and found that the existential dimension of spirituality was positively associated with health-related quality of life in Canada. In a recent study by Reig-Ferrer (2012) in hemodialysis patients, Spiritual well-being was significantly associated with quality of life variables, health status and personal happiness in patients on dialysis.

Although there are studies that support the positive relationship between spiritual well-being and psychosocial adjustment and quality of life spiritual well-being is not considered a common part of palliative and/or supportive medical care (Sulmasy, 2002). In addition, QoL could be related to culture and religion, but limited studies have evaluated the relationship between religious coping and quality of life among patients with ESRD in Muslim countries such as Iran. Therefore, the purpose of this study was to determine the relationship between spiritual wellbeing and quality of life in hemodialysis patients in Iran.

## 2. Methods

### 2.1 Design and Patients

This cross-sectional study was conducted from December 2012 to June 2013 in Qom, a holy city in the central region of Iran. The patients referred to main hemodialysis centers were selected using a convenient sampling method according to the following inclusion criteria:

- age 15 years and older;
- having a history of hemodialysis for more than six months;
- ability to communicate in Persian;
- having no previous psychiatric disease;
- not taking any psychoactive medicine;
- willingness to participate in this study.

The study procedure was explained to the patients who met the eligibility criteria and the various questionnaires used were collected by the principal researcher.

### 2.2 Instruments

For data collection, a three-part questionnaire was used as follows:

1). To collect clinical and socio-demographic information. The questionnaire consisted of questions concerning age, marital, economical and educational status, employment, comorbidity, duration and number of hemodialysis, as well as smoking and body mass index (BMI).

2). Health-related quality of life was measured using the Short-Form Health Survey (SF-36). The SF -36 has 8 subscales which include physical functioning, bodily pain, general health, vitality, social functioning, role limitations due to physical problems, role limitations due to emotional problems and mental health. This questionnaire also analyzes the physical and mental components defined by four domains each. The physical components are physical functioning, role limitations due to physical problems, bodily pain, and general health. The mental components are vitality, social functioning, role limitation due to emotional problems and mental health. Scores in each subscale range from zero to 100, with zero representing the worst conditions and 100 representing the best possible score. Previous evaluations of the original as well as the Persian version of SF-36 indicated good reliability and validity (Ware Jr et al., 1998; Montazeri et al., 2005).

3). Spiritual Well-Being was measured using the Spiritual Well-Being Scale (SWBS). It was developed by Paloutzian and Ellison (1982) as a measure to assess this aspect of a person’s spiritual life that transcends any particular religion. The instrument includes two components: a religious well-being subscale, which measures one’s relationship with God and assesses the vertical dimension of spirituality and an existential well-being subscale, which measures the horizontal dimension of well-being in relation to the world about us, life purpose, and life satisfaction. The SWBS is a 20-item questionnaire in which each item is rated on a six point Likert scale ranging from 1 to 6 with a higher number representing greater well-being (Paloutzian & Ellison, 1982). The Persian version of this questionnaire was validated by Rezaei et al. (2008).

### 2.3 Data Analysis

Descriptive statistics were used to explore the data. Independent t-test, one-way analysis of variance was used for comparison. Logistic regression analysis was performed to assess the relationship between spiritual well-being and health-related quality of life while controlling for socio-demographic and clinical variables. Data analysis was conducted with SPSS v.16 (SPSS, Inc). P-Value less than 0.05 was considered significant in all analyses.

### 2.4 Ethical Considerations

The study research proposal was approved by the deputy of research, Qom University of Medical Sciences. Ethical approval was granted by the Medical Ethics Committee that corroborated the ethical considerations through out the study. Participation in this study was voluntary and patients were thus free to withdraw from the study at any time without having any effect on their treatment process. All patients were included after informed consent.

## 3. Results

In all, 120 patients with ESRD were approached. Of these, 95 individuals agreed to participate in the study (response rate 79.1%). Mean and standard deviation of the patients’age was 50.4±15.72 years and 61.1 percent of the patients were male. The majority of the patients (76.8) were married. The time on hemodialysis was 37.83 months (SD=39.14 months). The main characteristics of the study participants are shown in Table 1.

**Table 1:** Clinical and socio-demographic information of the patients (N = 95)

	N	(%)
**Age (years)**		
Mean (SD)	50.40	15.72
**Gender**		
Males	58	61.1
Females	37	38.9
**Educational status**		
Illiterate	30	31.5
Primary school	30	31.5
High school	28	29.4
Secondary school	7	7.6
**Marital status**		
Single	12	12.6
Married	73	76.8
Divorced/widowed	10	10.6
**Employment status**		
Employed	21	22.1
Unemployed/housewife	74	77.9
**Accommodation**		
Urban	88	92.6
Rural	7	3.4
**Comorbidity disease**		
Yes	55	57.9
No	40	42.1
**Body mass index**		
Mean (SD)	20.16	3.11
**Economic status**		
Poor	45	47.3
Intermediate	40	42.1
Good	10	10.6
**Smoking status**		
Smoker	16	16.8
Non-smoker	79	83.2
**Time on dialysis (months)**		
Mean (SD)	37.83	39.14
**Dialysis number**		
Mean (SD)	2.92	0.30

The mean score of spiritual well being was 91.98 (SD=15.09) with religious and existential domains mean scores of 50.76 (SD=8.06) and 41.22 (SD=8.91) respectively. Age significantly correlated with spiritual well-being. One-way ANOVA showed a significant positive correlation between marriage and spiritual well-being (p=0.04).

Higher and lower scores were seen for social functioning (59.37±26.57), and role limitations due to physical health problems (30.91±35.15).

From demographic factors, only age had a significant negative relation with physical functioning. With regard to clinical variables, the number of hemodialysis sessions had significant correlation with physical functioning and role limitation (physical and emotional). As well, time on dialysis had a positive correlation with physical and emotional role limitations.

The results of Independent t-test showed there was a statistically significant difference between level of spiritual well being and some quality of life domains consisting of body pain, vitality, social function and mental health (Table 2). As well the Pearson correlation coefficient indicated a significant correlation between spiritual well being and bodily pain, vitality, social functioning and mental health domains (Table 3). It was a highly significant correlation between these two dimensions of spiritual well-being (religious well-being and existential well-being) (r=.74, p=.000) and also between each dimension and total score of spiritual well-being (r=.93, p=.000).

**Table 2a:** Mean and standard deviation of study variables

Quality of life scores	Mean	SD
Physical functioning	43.82	27.60
Role physical	30.91	35.15
Bodily pain	45.51	29.52
General health	39.54	19.76
Vitality	37.44	23.74
Social functioning	59.37	26.57
Role emotional	45.31	41.81
Mental health	47.23	23.23
Spiritual well-being	91.98	15.09
Existential well-being	41.22	8.91
Religious well-being	50.76	8.06

**Table 2b:** Mean and standard deviation of quality of life of the patients based on the spiritual well-being

Variable	Spiritual well-being (Mean ± SD)		P

	Moderate n=63	High n=29	
Physical functioning	43.01(26.72)	46.37(29.05)	0.58
Role physical	27.77(32.10)	37.93(39.87)	0.19
Bodily pain	41(28.14)	58.24(27.65)	0.008
General health	47.36(14.44)	52.94(17.13)	0.10
Vitality	29.52(18.02)	51.89(21.14)	0.0001
Social functioning	55.42(25.74)	70.25(23)	0.009
Role emotional	41.26(40.47)	58.62(41.45)	0.06
Mental health	41.65(22.17)	64.41(19.95)	0.0001

**Table 3:** Pearson correlations among quality of life and Spiritual well-being

Variables	Physical functioning	Role physical	Bodily pain	General health	Vitality	Social functioning	Role emotional	Mental health
Existential well-being	0.05	0.12	0.26*	0.13	0.36**	0.28**	0.23*	0.34**
Religious well-being	0.18	0.15	0.37**	0.15	0.49**	0.30*	0.24*	0.39**
Total Spiritual well-being	0.12	0.15	0.33**	0.14	0.45**	0.31**	0.25*	0.39**

** Correlation is significant at the 0.01 level

* Correlation is significant at the 0.05 level

Spiritual well-being subscales as well as the socio-demographic and clinical variables were entered in multiple logistic regression models as predictor variables. The results of multiple logistic regression model showed that religious well-being was associated with better QoL in both domains of physical (OR=1.17; p=0.01) and mental (OR=1.14; p=0.02) components after controlling for socio-demographic and clinical data (Table 4).

**Table 4:** Consideration of independent variables relationship with quality of life according to multiple logistic regression

Independent variables	Physical components			Mental components		

	OR	P	Interval confidence 95% CI	OR	P	Interval confidence 95% CI
**Socio-demographic**						
Age	.99	.86	.94-1.05	1.06	.82	.95-1.06
Gender	.71	.61	.18-2.67	1.10	.89	.28-4.27
Marital status	.31	.27	.03-2.52	. 27	.20	.03-2.08
Education	1.26	.36	.78-1.95	1.43	.13	.89-2.29
Economic status	1.67	.04	1.27-6.13	1.15	.47	.42-2.03
**Clinical**						
Time on dialysis	1.01	.07	.99-1.03	1.01	.08	.99-1.03
Number of dialysis	5.95	.14	.53-66.83	11.35	.06	.84-153.13
Current smoker	.38	.31	.05-2.50	1.70	.55	.29-9.75
Comorbidity	.66	.17	.36-1.19	.92	.66	.64-1.32
**Spiritual well-being**						
Existential well-being	.97	.65	.86-1.09	1.03	.60	.92-1.15
Religious well-being	1.17	.01	1.04-1.32	1.14	.02	1.02-1.29

## 4. Discussion

The aim of our research was to identify the relationship between spiritual wellbeing and quality of life in hemodialysis patients. According to our study results, spiritual well-being in both religious and existential domains were associated with bodily pain, vitality, social functioning and mental health. Meaning of life, feeling of belonging to a sublime source and hope to be supported by God are the ways which religious people use to suffer less stress during exposing stressful events in their life. Our study findings in are consistent with previous studies demonstrating the importance of spirituality in other populations facing advanced illness such as in breast cancer (Cotton et al., 1999), chronic pain (Rippentrop et al., 2005), and advance cancer (Vallurupalli et al., 2011) multiple sclerosis (Allahbakhshian et al., 2010), HIV (Dalmida et al., 2011), and chronic renal failure (Finkelstein et al., 2007). In a descriptive study, better coping with a disease, mainly in the psychosocial aspect, correlated with higher spiritual, existential, and religious wellbeing (Tanyi et al., 2007). Similarly, a qualitative study carried out by Walton assessed 11 patients on dialysis aiming at analyzing the meaning of spirituality for those patients and the way they used it to adapt to their new reality. Patients described spirituality as a life-giving force from within, full of awe, wonder, and solitude, that inspires one to strive for balance in life. (Walton. 2002). In 2002, Patel and Shah carried out a study at the George Washington University with 53 patients on hemodialysis and reported a direct association of the importance of spirituality and religious involvement with the following: social support; coping with the disease; and quality of life.

We found a highly significant correlation between two dimensions of spiritual well-being (religious well-being and existential well-being) which is like the other published papers on spiritual well-being and might be because of two reasons: religion-basis of existential beliefs of Iranian people and also the internal consistency of the instrument.

A multi-institutional survey-based study of cancer patients of all stages found that spirituality wellbeing was an independent predictor of QoL even after controlling for other key determinants of QoL (Brady et al., 1999). In another study, spirituality was recognized as a key factor among the patients chronically affected by mustard gas in the disaster of war in accepting and coping with their chronic illness complications (Ebadi et al., 2009). The latter results are congruent with the present study, i.e., according to the findings of the current study, religious well-being is associated with improved QoL in hemodialysis patients.

## 5. Conclusion

In conclusion, the findings from our study highlight the importance of spiritual well-being for QoL of hemodialysis patients. Religious wellbeing predicted better quality of life in patients with ERDS. Focus on spirituality reinforcement in routine patients care could improve different aspects of QoL. As well, spiritual support of family or charities could have a good impact in QoL of these patients.

Regarding the high correlation between religious wellbeing and spiritual wellbeing, religion basis of existential beliefs is concluded. So, it is recommended that religious organizations focus on spiritual support programs of patients with chronic diseases. As well, spiritual-therapy, as a context oriented complementary psychological intervention is recommended for such patients in Iran.

### Study Limitations

Several limitations of this research should be noted. This cross-sectional study did not allow for measurement of variables over time. Non-random sampling and limit size of sample reduce generalizability of the findings in this study and doing of study by a larger sample size can effective in upgrade of this limitation. Performing the triangulation and qualitative researches, in order to have better understanding of the effect of spirituality on health and the way to improve the quality of life in hemodialysis patients, is recommended.

### Authors’ Contribution

ZT was the main investigator, designed the study, collect the data, and wrote the first draft. HZ was the study supervisor and contributed to the writing process and Analysis. SFF contributed to the data collection and analysis and writing process. All authors read and approved the paper.
